# Drk1, a Dimorphism Histidine Kinase, Contributes to Morphology, Virulence, and Stress Adaptation in *Paracoccidioides brasiliensis*

**DOI:** 10.3390/jof7100852

**Published:** 2021-10-12

**Authors:** Caroline Maria Marcos, Haroldo Cesar de Oliveira, Patrícia Akemi Assato, Rafael Fernando Castelli, Ana Marisa Fusco-Almeida, Maria José Soares Mendes-Giannini

**Affiliations:** 1 School of Pharmaceutical Sciences, São Paulo State University (UNESP), Araraquara 14800-903, Brazil; marcos_caroline@yahoo.com.br (C.M.M.); haroldocdoliveira@gmail.com (H.C.d.O.); patricia.assato@gmail.com (P.A.A.); ana.marisa@uol.com.br (A.M.F.-A.); 2 Instituto Carlos Chagas, Fundação Oswaldo Cruz (Fiocruz), Curitiba 81350-010, Brazil; rafaelf.castelli@gmail.com; 3 Laboratório Central de Multiusuários, Faculdade de Ciências Agronômicas, Campus Botucatu, UNESP—Universidade Estadual Paulista, São Paulo 18610-034, Brazil; 4 Programa de Pós-Graduação em Biologia Parasitária, Instituto Oswaldo Cruz, Fundação Oswaldo Cruz (Fiocruz), Rio de Janeiro 21040-360, Brazil

**Keywords:** dimorphism regulating-histidine kinase, Drk1, *Paracoccidioides brasiliensis*, antisense-RNA silencing, cell wall, virulence

## Abstract

*P. brasiliensis* is a thermally dimorphic fungus belonging to *Paracoccidioides* complex, causative of a systemic, endemic mycosis limited to Latin American countries. Signal transduction pathways related to important aspects as surviving, proliferation according to the biological niches are linked to the fungal pathogenicity in many species, but its elucidation in *P. brasiliensis* remains poorly explored. As Drk1, a hybrid histidine kinase, plays regulators functions in other dimorphic fungi species, mainly in dimorphism and virulence, here we investigated its importance in *P. brasilensis*. We, therefore generated the respective recombinant protein, anti-PbDrk1 polyclonal antibody and a silenced strain. The Drk1 protein shows a random distribution including cell wall location that change its pattern during osmotic stress condition; moreover the *P. brasiliensis* treatment with anti-PbDrk1 antibody, which does not modify the fungus’s viability, resulted in decreased virulence in *G. mellonella* model and reduced interaction with pneumocytes. Down-regulating PbDRK1 yielded phenotypic alterations such as yeast cells with more elongated morphology, virulence attenuation in *G. mellonella* infection model, lower amount of chitin content, increased resistance to osmotic and cell wall stresses, and also caspofungin, and finally increased sensitivity to itraconazole. These observations highlight the importance of PbDrk1 to *P. brasiliensis* virulence, stress adaptation, morphology, and cell wall organization, and therefore it an interesting target that could help develop new antifungals.

## 1. Introduction

The capacity to survive inside the mammalian host is a feature that is undoubtedly a condition that requires a radical change to pathogens that live in the host environment [1]. The morphogenetic adaptation known as dimorphism allows both, i.e., growth in the environment and host colonization, critical for the lifecycle of dimorphic fungi [2,3]. Among the dimorphic fungi, six phylogenetically related ascomycetes genus in the order Onygenales, *Blastomyces*, *Histoplasma*, *Coccidioides*, *Sporothrix*, *Talaromyces,* and *Paracoccidioides,* change their morphologies once spores are inhaled and reach the lungs (or at 37 °C), from mycelia in the environment temperature to pathogenic yeasts form [1].

*Paracoccidioides* spp. are the most important to Latin America due to the endemic aspects of paracoccidioidomycosis (PCM), a life-threatening invasive disease caused by this genre [4,5]. The dimorphic nature of *Paracoccidioides* spp. has been well-known for decades, however, the molecular mechanisms related to the dimorphism process have not been fully elucidated. The morphological changes from the mycelium to the yeast form are essential for the development and establishment of disease in the host. These changes are related to the cell wall, that it can act as a shield to prevent host recognition, and essential to developing a protective immune response [6] that can significantly contribute to infection control [7].

The detection of specific environment signals occurs through signaling pathways [8] and can alter gene expression and protein synthesis, changing the content and architecture of the cell wall [9]. Comprehension of fungi management to perceive and process abiotic and biotic factors contributing to their interaction with the environment is of great importance, specially the two-component signaling systems that can be involved in countless aspects as biological roles, general responses to different types of stress, metabolism, morphological switches, and pathogenicity [10].

The Hog1 MAP kinase belonging to the HOG (high-osmolarity glycerol) pathway, senses and responds to both osmotic and oxidative stresses, adapting the fungus to these unfavorable conditions; furthermore, it acts in fungal homeostasis, regulating the cell wall biosynthesis [11].

The two-component signal transduction system (TCST) comprises a hybrid histidine kinase, a protein that has both, histidine kinase and receiver domains [12] that senses environmental stimulus and leads to the autophosphorylation on histidine residues, afterward a phosphoryl transfer reaction to the receiver domain on aspartate residues; the latter acts like a transcription factor activating several genes related to stress adaptation, spore formation, cell–cell communication, virulence factors, and others [13].

Drk1 (dimorphism regulating histidine kinase 1) is considered a variant of the TCS, it is expected to be part of the HOG signaling pathway, facilitating the fungi adjustment to “stressful” conditions, as oxidative, osmotic, and temperature oscillations [14], but the exact stimulus that Drk1 senses still needs to be investigated.

Drk1 is described as a homolog to Sln1 in *Saccharomyces cerevisiae* [15]. The hybrid kinase components can be encoded by *DRK1* and a homolog to SLN1 in most dimorphic fungi, and in some cases have both SLN1 and *DRK1*, in which the importance and role of each one can vary according to the species. According to Catlett et al. [16] that described six groups of HHKs (hybrid histidine kinases), Sln1 is classified in the transmembrane group of VI HHKs [16,17] and Drk1 belongs to the group III of HHKs and seems to be associated with the MAPK cascade, regulating the adaptation to osmotic conditions through Hog1 activation [18,19].

Nemecek et al. [1] provide a piece of evidence to the morphological transition regulation through a screening of insertional mutagenesis for regulators of the *BAD1* (*Blastomyces* adhesin-1), a yeast phase-specific gene of *Blastomyces,* where they identified the Drk1, a transmembrane hybrid histidine kinase, that besides acting in the control of dimorphism also govern the virulence control in *B. dermatitidis* and *Histoplasma capsulatum.*

DRK1-knockdown confers to *B. dermatitidis* and *H. capsulatum* impairment in the mycelium to yeast conversion, forming only pseudohyphae. The silenced strains showed a reduced amount of 1,3 α-glucan, aberrant chitin distribution, increased sensibility to cell wall stressors, defects related to the expression of important virulence factor, as well as virulence attenuation [1].

*Talaromyces* (*Penicillium*) *marneffei* has both, DrkA and SlnA homologs to Drk1 and Sln1, respectively. Boyce et al. [20] using a single and double-deletion mutants demonstrated a partial overlap and single roles on the growth and development of the fungus. The osmotic stress adaptation and yeast grown in vivo requires both *DRKA* and *SLNA,* and both deletion results in abnormalities in the cell wall; moreover, SlnA is required for conidia germination and DrkA to dimorphism switching during macrophage infection.

*Sporothrix schenckii* demonstrated an increased expression of DRK1 in the yeast-phase [21] and its silencing resulted in alterations of cell wall composition and integrity, melanin synthesis, yeast-cell phase formation, and transcription of *STE20*, a gene related to the HOG pathway, that together also alters the fungal pathogenicity in a murine model [22].

In *P. brasiliensis,* the signaling pathways that govern morphological conversion and pathogenic abilities remain very little elucidated. Chaves et al. [23] demonstrated a high expression of Drk1 transcripts by qPCR in the yeast and the transition to mycelium form in a high virulent isolate of *P. brasiliensis* obtained after passage into the animal, suggesting its importance to the morphology and virulence of this fungus. Drk1 seems to be involved in several aspects as fungal response to changes in the environment, leading to consequences like cell wall alterations and dimorphic transition, which together impact the fungal infection by dimorphic fungi.

Exploring the cell signaling pathways in virulent fungi is important due to their implications in pathogenicity regulation [24]. Therefore, to better understand the roles of Drk1 in *P. brasiliensis* we employed different molecular approaches as recombinant protein, antibody, and a silenced strain construction to evaluate its localization, influence on morphology, virulence, cell wall organization, and stress adaptation. 

## 2. Materials and Methods

### 2.1. P. brasiliensis Isolate and Culture Conditions

*P. brasiliensis* isolate (Pb18) was maintained on solid Fava-Netto medium before the assays were cultivated in liquid brain-heart-infusion (BHI) medium supplemented with 1% glucose in a shaker under 150 rpm of agitation for 72 h at 37 °C. The number of viable yeast cells was determined using a dye exclusion test with trypan blue [25], and cell suspensions were used with viability greater than 90%. Tests with microorganisms were carried out in the class III biosafety cabinet.

### 2.2. Vector Construction, Heterologous Expression of PbDrk1 Recombinant (rPbDrk1), and Purification

RNA sample isolated from *P. brasiliensis* yeast cells using TRIzol^®^ reagent (Invitrogen Life Technologies, Carlsbad, CA, USA) according to the supplier specifications was transcribed to cDNA using RevertAid H Minus Reverse Transcriptase (Fermentas, Waltham, MA, USA). Initially, oligonucleotides (Table 1) containing *Nde*I and *Not*I restriction sites, were constructed to amplify the 3879-bp cDNA of PbDRK1 (PADG 07579) by polymerase chain reaction (PCR). PCR fragment was ligated in plasmid pGEM-T-Easy and used to transform *E. coli* DH5α cells. Then, DRK1 fragment was cut out from pGEM-T-Easy using *Nde*I and *Not*I (Fermentas, Waltham, MA, USA) and subcloned in pET28a vector using the same restriction sites. The pET28/*DRK1* construct was transformed into *E. coli* Rosetta. Transformants were selected on Luria-Bertani (LB) plates (1% tryptone, 0.5% NaCl, 0.5% yeast extract, 1.5% agar, pH 7.5) containing 30 µg mL^−1^ kanamycin and chloramphenicol (34 µg mL^−1^). The recombinant plasmid pET28/*DRK1* was verified by PCR using oligonucleotides DRK1-F and DRK1-R (Table 1) and by restriction diagnostic through digestion with *Nde*I and *Not*I. The PCR parameters were an initial denaturation 94 °C for 4 min, 25 cycles at 94 °C for 1 min, annealing at 55 °C for 1 min, extension at 72 °C for 1 min, and a final extension at 72 °C for 7 min. The PCR and restriction fragments were separated and analyzed on 0.8% (*w*/*g*) agarose gel electrophoresis.

Single colonies were grown to log phase (OD_600nm_ of 0.5–0.8) at 220 rpm, 37 °C in a shaker incubator in the selective medium previously described, and then gene induction was done using 0.4 mM of isopropyl-β-D-thiogalactopyranoside (IPTG; Sigma-Aldrich, Saint Louis, MO, USA) for different incubation periods and temperatures, 1, 3, and 24 h at 37 and 30 °C (220 rpm). For the purification of rPbDrk1, after IPTG induction bacterial cells were harvested and suspended in lysis buffer containing 10 mM Tris-HCl pH 8.0, 1 mM phenyl methyl sulfonyl fluoride, and 10 mg mL^−1^ lysozyme at 4 °C for 30 min, disrupted by sonication of 3 cycles of 2-min pulse and then centrifuged at 10,000 rpm for 45 min. The clarified lysate was chromatographed using HisTrap FF crude column (GE Healthcare, Chicago, IL, USA) followed by passing the eluted fractions in Centricon filters 100 kDa (Merck-Millipore, Burlington, MA, USA), both according to the manufacturer’s specifications. The expression induction of rPbDrk1 as well as its purification were evaluated by 12.5% SDS-PAGE (sodium dodecyl sulfate-polyacrylamide gel electrophoresis) [26] and gel-protein detection by Coomassie Brilliant Blue R350 staining. Protein concentration was determined using the Bradford protein assay (Bio-Rad, Pleasanton, CA, USA).

### 2.3. Mass Spectrometric Analysis of rPbDrk1

SDS-PAGE was carried out with the rPbDrk1 protein. The assumed recombinant protein band was cut out from the gel and subjected to sample preparation as described by Marcos et al. [29] to then analyzed by LC-MS/MS to confirm the protein identity. Extracted peptides were analyzed with amaZon SL ion trap mass spectrometer (Bruker Daltonics, Billerica, MA, USA) connected to a UFLC system (SHIMADZU-Nexera XR, Kioto, Japan) under control of HyStar software (Bruker Daltonics, Billerica, MA, USA) data acquisition. LC-MS/MS data files were converted to a mascot generic file format using DataAnalysis software (Bruker Daltonics, Billerica, MA, USA). MS data processing was performed with Proteinscape software (version 3.1, Bruker Daltonics, Billerica, MA, USA) using the Mascot algorithm (v2.3, Matrix Science, London, UK) for database searching configured with UniProtKB/Swiss-Protdatabase (https://web.expasy.org/docs/swiss-prot_guideline.html) 28 February 2018.

### 2.4. Antibody Production

To prepare the antigen for animals’ immunization, the target protein band was removed from the SDS-PAGE gel and separated from the polyacrylamide matrix by electroelution (Bio-Rad, Pleasanton, CA, USA). Male New Zealand White rabbits (3 month’s age) were immunized intradermally with 1 mL of equal mixture of rPbDrk1 electroeluted purified protein and Complete Freund’s adjuvant. Eight subsequent immunizations, spaced every 3 days, using rPbDrk1 in an emulsion incomplete Freund´s adjuvant (ratio 1:1) were given. Rabbits were exsanguinated by cardiac puncture 10 days after the last booster and the sera were purified with ammonium sulfate precipitation (1.56 M, pH 5.6), and stored at −20 °C [29]. To be used as negative control, pre-immune serum was collected before the first immunization and stored under the same conditions.

### 2.5. Protein Extraction, SDS-PAGE, and Western Blotting

*P. brasiliensis* yeast cells were cultured for 3 days in BHI medium plus 1% glucose at 37 °C (150 rpm) for protein extraction. After the growth, cells were washed twice with cold, sterile water (5000 rpm, 5 min, 4 °C). The resulting pellet was suspended in extraction buffer (10 mM Tris-HCl, pH 8.8) containing a cocktail of proteases inhibitors, 1 mM PMSF (phenyl methyl sulfonyl fluoride), and 1X PLAAC (pepstatin, leupeptin, aprotinin, antipain, chymotrypsin). An equal amount of glass beads was mixed to the samples and submitted to disruption alternating vortex and liquid N_2_ bath for 15 min (and eventually ice to avoid sample heating). Protein extract was centrifuged at 14,000 rpm for 15 min, and the supernatant was stored at −80 °C until use. Bradford method was employed to determine the protein concentration [30].

For Western blotting, both total protein extract from *P. brasiliensis* and the rPbDrk1 protein were separated in 12.5% SDS-PAGE [26], wet transferred to nitrocellulose membranes overnight at 4 °C [31] and blocked with 5% skimmed milk in PBS 0.5% Tween20 (PBS-T) for 2 h. Membranes were incubated overnight with primary antibodies, anti-PbDrk1, and pre-immune antisera at different dilutions (1:50, 1:100, and 1:250) in blocking buffer, washed three times with PBS-T, followed by incubation with, anti-rabbit IgG-HRP antibody (Sigma-Aldrich, Saint Louis, MO, USA) in 1:2000 dilution for 2 h at room temperature (RT). Finally, the membranes were washed three times with PBS-T, and immune complexes were visualized employing 3,3′-diaminobenzidine (Sigma-Aldrich, Saint Louis, MO, USA) in the presence of H_2_O_2_.

### 2.6. Protein Localization: Indirect Immunofluorescence and Immunogold

To the confocal analysis, yeasts cells of *P. brasiliensis* cultivated for three days in BHI medium supplemented with 1% glucose, 37 °C (150 rpm) were separated in 10^6^ yeast cells mL^−1^ suspensions and growth in two conditions: (a) without stress (BHI medium) and (b) with stress in BHI medium containing 0.15 M NaCl for 1 h (37 °C, 150 rpm). After the incubations, cultures were centrifuged, washed with PBS, fixed with 4% paraformaldehyde for 1 h, and the cells were permeabilized by incubating for 45 min at RT with 1% Triton-X 100. Sedimented cells were washed three times with PBS, followed by the block with bovine serum albumin (BSA) 3% (*w*/*v*) in PBS for 1 h at 37 °C, and then incubated with the anti-PbDrk1 antibody (20 µg mL^−1^ in blocking solution) overnight at 4 °C. After 3 × 10 min washes, cells were incubated with 10 µg mL^−1^ of the anti-rabbit-IgG Alexa-594 antibody (ThermoFisher, Carlsbad, CA, USA) in blocking buffer for 1 h at 37 °C. Finally, the cells were incubated with calcofluor white solution (50 µg mL^−1^) (Sigma-Aldrich, Saint Louis, MO, USA) in PBS for 1 h at RT, washed five times, and observed using the confocal microscope Carl Zeiss LSM 800 with Airscan (School of Dentistry of Araraquara, FOAr/UNESP, Araraquara, São Paulo, Brazil); the images were processed using the Software ZEN BLUE 2.3 System. A control with secondary antibody alone was performed to determine whether the secondary antibody directly binds the sample.

To the immunogold, 1 × 10^8^ mL^−1^ yeast cells of *P. brasiliensis* were fixed overnight at 4 °C in fixative containing 2.5% glutaraldehyde in 0.1 M sodium cacodylate buffer, pH 7.2 and then sections collected on nickel grids were prepared by the electron microscopy service of the Institute of Biomedical Sciences—ICB-I, USP. The sections were blocked in buffer consisting of 1.5% (*w*/*v*) BSA and 0.05% Tween-20 (*v*/*v*) in PBS (BSA/Tween 20 blocking solution), and then incubated overnight at 4 °C with anti-PbDrk1 antibody diluted 1:50 (40 µg mL^−1^) in BSA/Tween 20 blocking solution. After washing with the same blocking solution, the grids were incubated with goat anti-rabbit IgG conjugated with 10 nm colloidal gold particles (Sigma-Aldrich, Saint Louis, MO, USA) diluted 1:10 in BSA/Tween 20 solution for 3 h at 4 °C [29]. Samples were examined using a Jeol-JEM-100 CXII (80 kV) transmission microscope (Jeol, Tokyo, Japan) in the Multiuser Laboratory of Electron Microscopy (LMME-FMRP/USP). Negative control was performed by reacting samples with the rabbit pre-immune serum (NRS).

### 2.7. P. brasiliensis Viability after the Anti-PbDrk1 Treatment

For testing the effect of the antibody on the viability of *P. brasiliensis*, 200 µL of 10^6^ yeasts mL^−1^ in BHI medium plus 1% glucose were added to each well of a 96-well plate. The anti-PbDrk1 and pre-immune antisera were both added to a final concentration of 20 µg mL^−1^. The plates were incubated at 37 °C at 150 rpm, and the viability was assessed by tetrazolium salt (XTT) (Sigma-Aldrich, Saint Louis, MO, USA) reduction assay as described by Meshulam et al. [32]. As for viability control, *P. brasiliensis* without antibodies treatment was employed.

### 2.8. Inhibition Assays with Anti-PbDrk11 Antibody and Consequences to P. brasiliensis-Pneumocytes Interaction by Flow Cytometry

*P. brasiliensis* yeast cells were treated with 20 µg mL^−1^ of anti-PbDrk1 and pre-immune antibodies, separately, at 37 °C, 150 rpm for 1 h, and then washed with PBS. The yeast cells were stained with FITC (500 µg mL^−1^) for 10 min at the same conditions, washed, and the inoculum adjusted to 10^6^ yeast cells mL^−1^ in Dulbecco′s modified Eagle medium (DMEM). The inocula were used to infect the pneumocytes (A549 cells) monolayer (at 95% of confluence, confirmed my microscopy) in a multiplicity of infection of 1:2, that was previously formed in 96-well plate after 24 h of incubation at 36.5 °C, 5% CO_2_ in DMEM enriched with 10% of fetal bovine serum (FSB). The infection was incubated under the same conditions described above for 2, 5, and 8 h. Then, non-adherent yeast cells were removed through washing three times with PBS, and the attached cells were pulled out from the plates with cold PBS and a mechanical detachment with a rubber scraper, fixed with 4% paraformaldehyde, and then analyzed with FACSCanto^TM^ (BD Biosciences, San Jose, CA, USA) cytometer using FACSDiva software [33]. To adjust the gates uninfected A549 cells and yeast cells, labeled and unlabeled, were employed. The percentage of A549 cells infected by adhered and/or internalized yeast cells was defined from FITC-positive population from the A549 gate within 10,000 acquired events.

### 2.9. Effect of the P. brasiliensis Anti-PbDrk1 Antibody Treatment on G. mellonella Infection

As well performed to the A549 cells assay, *P. brasiliensis* was treated with anti-PbDrk1 and pre-immune serum, and then 5 × 10^6^ yeast cells (in PBS) were used to infect *G. mellonella* (weighing 0.15–0.2 g) using Hamilton syringe (Hamilton, Reno, NV, USA) through the last proleg. To observe possible consequences related to physical injury on the larvae survival, a control group was injected with PBS alone. The larvae were maintained at 37 °C and the mortality recorded daily for one week [34].

### 2.10. PbDRK1 Gene Silencing with Antisense Molecules and Agrobacterium Tumefaciens Transformation (ATMT)

Antisense RNA (aRNA) and ATMT were employed to generate a DRK1-silenced *P. brasiliensis* strain [35,36]. Briefly, three sequences of different coding regions of the DRK1 gene comprising nucleotides between positions 112–230 (AS2), 231–333 (AS3), and 320–481 (AS4) were amplified using a high-fidelity DNA polymerase (Platinum^TM^, ThermoFisher, Carlsbad, CA, USA) and primers DRK1-AS2-F/R, DRK1-AS3-F/R, and DRK1-AS4-F/R (ThermoFisher, Carlsbad, CA, USA) (Table 1). The inserts AS2, AS3, and AS4 were ligated individually into the pCR35 vector under the control of the calcium-binding protein (CBP-1) promoter. The pCR35 vectors containing AS2, AS3, and AS4 were subcloned into the pUR5750 parenteral binary vector and used to transform *A. tumefaciens* LBA1100 via electroporation. After kanamycin (100 µg mL^−1^) resistance selection and confirmation by PCR, positive *A. tumefaciens,* for pUR5750 transformation were employed in the *P. brasiliensis* yeast cells transformation through co-cultivation of both microorganisms in a 6-well culture plate at a 1:1, 1:10, and 1:100 ratios on liquid induction medium (IM) at 25 °C for 72 h at agitation speed of 100 rpm according to Goes et al., [28] with modifications. After co-cultivation, 2 mL of BHI supplemented with 1% glucose (BHID), 10% of FSB, and 200 µg mL^−1^ of cefotaxime, was added and the cocultivation was incubated for another 48 h at 36 °C (150 rpm) to promote the expression of the hygromycin B resistance (hph) gene and to eliminate *A. tumefaciens* cells. Subsequently, the cultures were centrifuged, the pellet cells suspended in 200 µL of the residual IM and plated on BHID, 5% (FSB), 100 µg mL^−1^ cefotaxime, and 100 µg mL^−1^ hygromycin. The plates were incubated at 36 °C until the growth of *P. brasiliensis* colonies (20–25 days). Isolated colonies of *P. brasiliensis* transformants were transferred to liquid BHID, 5% FBS, 100 µg mL^−1^ cefotaxime, and 100 µg mL^−1^ hygromycin in 12-well culture plate and subcultured for three successive rounds of 3 days to select only stable integrants and remove remaining untransformed yeast cells. Then, *P. brasiliensis* transformants were transferred to non-selective medium BHID, 5% FSB and subcultured for three more successive rounds to inspect mitotic stability followed by the analysis of growth capacity in a 12-well culture plate with selective liquid BHDI, 5% FBS and 100 µg mL^−1^ of hygromycin.

### 2.11. PCR Detection of Hygromycin Resistance Gene (hph) and Analysis of PbDRK1 Transcript Levels by qRT-PCR (Quantitative Real Time PCR)

First, we evaluated the insertion and retention of T-DNA in the hygromycin resistant colonies of *P. brasiliensis* by direct PCR as described by Goes et al. [28]. The PCR detection of hph gene was carried out with a 50 µL reaction mixture consisting of 5 µL of cell lysate, 0.4 µM of primers hph-F/R (Table 1), 1.25 units of Taq polymerase, 0.2 mM dNTP, 2 mM MgCl_2_, and Taq buffer. The PCR reactions were run with cycling protocol of 4 min at 94 °C, followed by 35 cycles of 30 s at 94 °C, 1 min at 52 °C, 2 min at 72 °C, and final extension for 10 min at 72 °C.

After detecting the hph gene in the *P. brasiliensis*-transformants, the DRK1 gene expression was determined by qRT-PCR. RNA was extracted from yeast cells of PbWT (*P. brasiliensis* wild-type), PbEV (*P. brasiliensis* transformed with empty vector), Pb*DRK1* aRNA2, Pb*DRK1* aRNA3, and Pb*DRK1* aRNA4. *P. brasiliensis* transformed with aRNA cassettes AS2, AS3, and AS4, respectively, using TRIzol^®^ (Invitrogen Life Technologies, Carlsbad, CA, USA) and glass beads maceration was treated with DNAseI (ThermoFisher, Carlsbad, CA, USA) after RNA purity assessment. The cDNA was synthesized from 1 µg of RNA using the RevertAid^TM^ H Minus Reverse Transcriptase (Fermentas, Waltham, MA, USA). Real-time PCR was carried out using the QuantiNova SYBR Green PCR kit with ROX, according to the manufacturer (Qiagen, Hilden, Germany). The 7500 Cycler Real-Time PCR System (Applied Biosystems^®^, Whaltham, MA, USA) was employed to the qRT-PCR assay. The sequences of the DRK1-qPCR-F/R primers used to detect the DRK1 mRNA expression levels are shown in Table 1. qRT-PCR melt curve was assessed to verify the single peaks [37]. Data were analyzed using the 2^−ΔΔCt^ method [38] using L34 gene (primers in Table 1) as endogenous control, and the target gene expression levels in PbWT and PbEV as calibrators.

### 2.12. Phenotypic Effects of PbDRK1 Silencing

#### 2.12.1. Growth Curve and Cell Viability

Inoculum of the strains, PbWT, PbEV, Pb*DRK1* aRNA2, Pb*DRK2* aRNA3, and Pb*DRK1* aRNA4 were adjusted to start the growth curve with 10^5^ cells mL^−1^ in 100 mL of BHI liquid medium with 1% glucose at 37 °C (150 rpm). Growth was assessed by spectrophotometric analysis (OD_600nm_; Epoch2, BioTek, Winooski, VT, USA) at 24 h intervals for 7 days. At times of 0 and 168 h, a sample was taken for viability assay with trypan blue dye, according to Marcos et al. [39].

#### 2.12.2. Morphological Analysis

Yeast cells of PbWT, PbEV, Pb*DRK1* aRNA2, Pb*DRK1* aRNA3, and Pb*DRK1* aRNA4 were grown under standard conditions as mentioned above. Then, the cells were washed with PBS and then fixed with 2.5% glutaraldehyde in a 0.1 M sodium cacodylate buffer (pH 7.2) for 1 h at 25 °C. After washing with sodium cacodylate buffer containing 0.2 M sucrose and 2 mM MgCl2, the cells were seeded on poly-D-lysine (0.01%) and covered with coverslips and incubated for 30 min at 25 °C. A series of gradual ethanol dehydration (30, 50, and 70% for 5 min, 90% for 10 min, and 100% twice for 10 min) was performed followed by a critical-point drying (LEICA EM CPD300, Wetzlar, Germany), and mounting on metallic bases to be covered with a gold layer [40]. Images were obtained using a scanning electron microscope (JEOL JSM-6010 Plus/LA) operating at 5 keV.

#### 2.12.3. Virulence Assay

To evaluate the effects of the DRK1 silencing in *P. brasiliensis*-virulence, we employed the *G. mellonella* model of infection with PbWT, PbEV, and Pb*DRK1* aRNAs strains. The infection was performed as described above.

#### 2.12.4. Chitin Level

All strains were cultured for 72 h at 37 °C (150 rpm) in BHI medium with 1% glucose. Then, aliquots of each strain were fixed with 4% paraformaldehyde, and the cell concentration was adjusted to 10^6^ cells mL^−1^ after PBS-washing. The suspensions were stained with 50 µg mL^−1^ calcofluor white (CFW) (ThermoFisher, Carlsbad, CA, USA), a specific chitin dye, for 1 h at RT. After staining, the yeast cells were washed three times, and the cell suspension was placed on a glass slide and observed under a fluorescent microscope DMi8 (Leica, Wetzlar, Germany), with the same laser exposure time, coupling with the acquisition image system LasAF. The fluorescence intensity (at least 50 yeast cells) was determined using ImageJ, and the results expressed as a mean of fluorescence intensity/area of the yeast cell.

#### 2.12.5. Susceptibility to Antifungals

Strains were tested for their antifungal susceptibility to itraconazole (ITZ) (Sigma-Aldrich, Saint Louis, MO, USA) and caspofungin (CSP) (Sigma-Aldrich, Saint Louis, MO, USA) according to CLSI (Clinical and Laboratory Standards Institute) document M27-A3 with some modifications [41]. The inocula containing 10^6^ cells mL^−1^, prepared with a 4-days culture in Fava-Netto medium at 37 °C, were diluted 1:50 in PBS and then 1:20 in RPMI 1640 medium (Gibco; ThermoFisher Scientific, Carlsbad, CA, USA) supplemented with 2% glucose (RPMI-2% gluc), giving a final concentration of 0.5 to 2.5 × 10^3^ cells mL^−1^ after antifungals addition. ITZ and CSP were prepared in RPMI-2% gluc. The final concentrations of ITZ and CSP varied from 0.0000625 to 0.032 µg mL^−1^ and 0.0625 to 32 µg mL^−1^, respectively. The plates were incubated for 48 h at 37 °C (150 rpm) and the viability was assessed using Alamar Blue (BioSource International, Camarillo, CA, USA), which has been added and incubated in the same conditions for 24 h [41]. The viability was assessed through absorbance evaluation at 570 and 600 nm.

#### 2.12.6. Susceptibility to Cell Wall Stresses

NaCl and Congo Red sensibility were performed through spot assay [42]. Inocula containing 107 cells mL^−1^ were prepared with strains grown for 4-days in Fava-Netto medium at 37 °C, and then undiluted culture and a ten-fold serial dilution of culture were spotted on YPD plates supplemented with Congo red (2.5 and 5.0 µg mL^−1^; CR) and NaCl (0.05 and 0.15 M), respectively. After incubation at 37 °C for 7 days, the plates were photographed.

### 2.13. Statistical Analysis

The statistical analysis was made using GraphPad Prism 5.0 (GraphPad software, Inc., La Jolla, CA, USA). One-way ANOVA followed by Tukey´s test and Student´s t-test were used to calculate the statistical significance. Survival curves were analyzed by Logrank test, and *p*-values of <0.05 were considered statistically significant.

## 3. Results

### 3.1. Molecular Cloning of cDNA Encoding a DRK1 of P. brasiliensis, Expression and Recombinant Protein Purification

The PbDrk1 was cloned into pET28a vector, and the construct generated, pET28/*DRK1,* was used to transform *E. coli* Rosetta. Colony-PCR and double restriction analysis using the target-specific primers and *Not*I/*Nde*I enzymes, respectively confirmed the success of transformation with the clones carrying the insert of the size expected for the target Drk1 (3879 bp), as well two fragments after digestion: ∼5.4 kb representing the pET28a vector and a ∼3.9 kb fragment representing DRK1 fragment.

**Figure 1:**
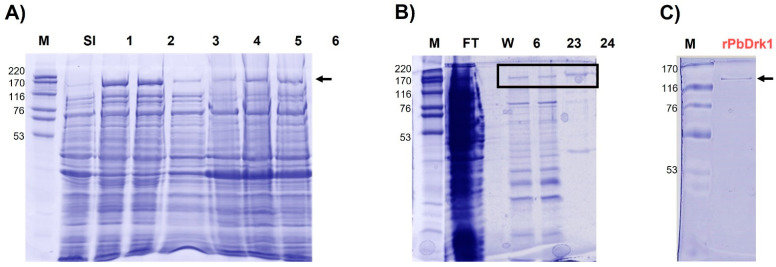
PbDrk1 expression and purification. (**A**) Expression of PbDrk1 in *Escherichia coli* Rosetta cells transformed with pET28/DRK1. Absence of IPTG (Lane SI); lanes 1 to 3: upon IPTG induction after 1, 3, and 24 h at 37 °C. Lanes 4 to 6: upon IPTG induction after 1, 3, and 24 h at 30 °C. (**B**) Protein purification after ion metal affinity chromatography (IMAC). SDS-PAGE of fractions from flow through (FT), wash (W), and 6, 23, and 24 elution fractions. (**C**) rPbDrk1 purified after affinity chromatography and passing by Centricon (100 kDa). Protein bands were revealed with Coomassie Blue staining. Lane M: standard protein marker (GE Healthcare, Chicago, IL, USA, 53–220 kDa).

A soluble fraction obtained from cells grown in the presence of 0.4 mM IPTG expressed a 142.5 kDa polypeptide with a higher expression at 37 °C from 1 and 3 h of IPTG induction (Figure 1A). The condition established in the small-scale expression, 0.4 mM of IPTG at 37 °C by 1 h, was employed in a large-scale production (1 L), and solubilized proteins were purified. It was possible to obtain a fraction containing the rPbDrk1 and a small number of contaminant proteins (Figure 1B), which have been successfully removed passing the sample in Centricon-filter (100 kDa), allowing the rPbDrk1 purification (Figure 1C).

The single band representing the purified protein was submitted to mass spectrometric identification to confirm the identity of the rPbDrk1 and detailed results are shown in Figure S1. The rPbDRK1 protein presented a molecular mass of 142.5 kDa and an acidic isoelectric point (pI) of 5.3. Overlapping peptides identified by mass spectrometry and amino acid sequence of PbDrk1 were possible to demonstrate a covering of 59% of the Drk1 protein (Figure S1).

### 3.2. Anti-PbDrk1 Polyclonal Antibody Recognizes the rPbDRK1 and Native Protein in P. brasiliensis

**Figure 2:**
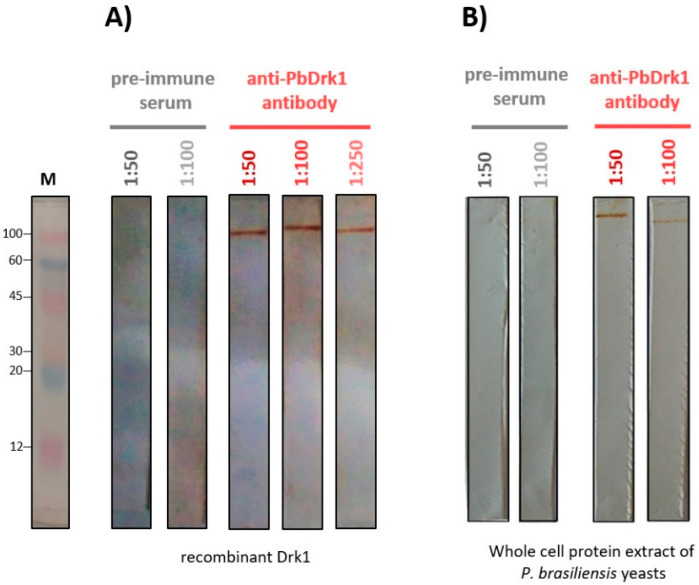
Western blotting of recombinant Drk1 and *P. brasiliensis* proteins recognized by anti-Drk1 antibody. Western blotting of (**A**) recombinant and (**B**) native Drk1 of *P. brasiliensis*. The non-specific reaction was evaluated using a pre-immune serum. M: standard protein marker (ColorBurst^TM^ Sigma-Aldrich, Saint Louis, MO, USA; 8–220 kDa). The molecular mass is indicated on the left in kilodaltons (kDa).

An anti-PbDrk1 rabbit polyclonal antibody was obtained through immunizing rabbits with PbDrk1 recombinant protein electro-eluted from the gel. As demonstrated in Figure 2, a single protein band on Western blotting was revealed in preparation with rPbDRK1 (Figure 2A) and the total protein extract of *P. brasiliensis* (Figure 2B) showing that the antibody was able to recognize the native protein of *P. brasiliensis.* The rabbit pre-immune serum did not react with all the samples (Figure 2A,B).

### 3.3. PbDrk1 Localization in the Yeast Cell Surface, Showing an Altered Pattern of Expression during Osmotic Stress Condition

**Figure 3:**
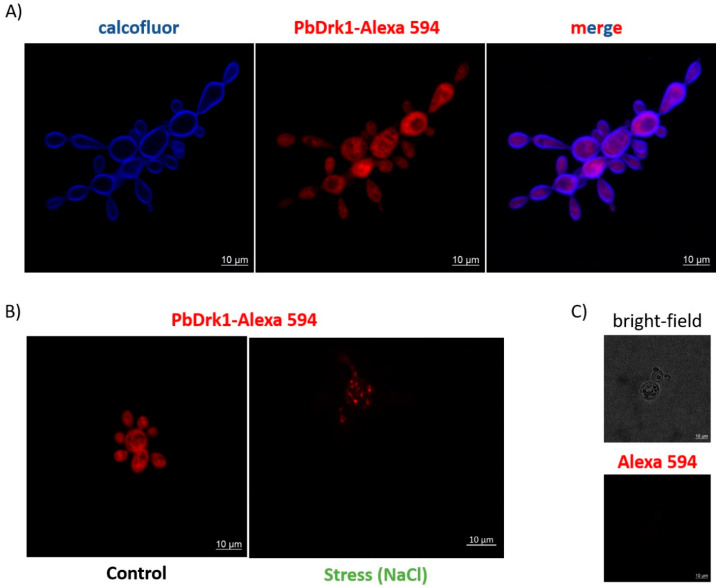
PbDrk1 localization and altered pattern of expression in osmotic stress condition. (**A**) Confocal microscopy of yeast cells. In blue: calcofluor white staining of the cell wall. In red: reactivity with primary anti-PbDrk1 antibody and anti-rabbit IgG-Alexa 594. (**B**) Confocal analysis under stress condition (0.15 M NaCl) and (**C**) control with secondary antibody alone. Alexa 594: secondary antibody anti-rabbit IgG conjugated with Alexa 594 nm.

The confocal microscopy images seen in Figure 3A show homogeneous fluorescence labels throughout the cell, both in the mother cells and buds, with an apparent co-localization of PbDrk1 with calcofluor white stain in the fungi cell wall. Once histidine kinases typically comprise the first sensing proteins of multistep phosphorelay signaling pathways osmosensing environmental alterations as osmotic stress, we evaluated the Drk1 expression during this condition. The hyperosmotic stress, with a sub-lethal concentration of NaCl, leads to a modification of the homogeneous distribution of Drk1 throughout the cell to a clustered accumulation, with a punctuate staining pattern in the cytosol (after 1 h of 0.15 M NaCl treatment, a subinhibitory concentration determined through spot assay) (Figure 3B).

**Figure 4:**
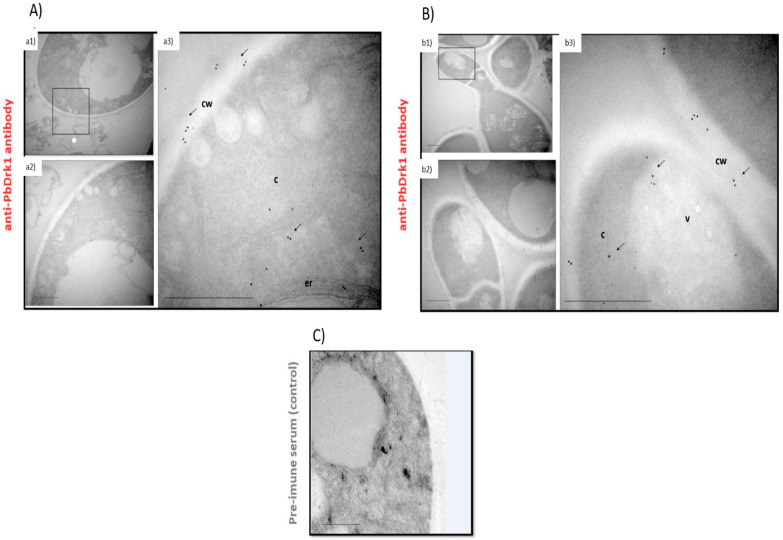
Immunogold analysis of PbDrk1 protein localization in *P. brasiliensis* yeast cells. The highlighted square in (**a1**) and (**b1**) points to the *P. brasiliensis* cell section examined in the differents microscope objectives (**a2**,**a3**,**b2** and **b3**). (**C**) Negative control with non-immune rabbit serum showed no labeling. Abbreviations: cw: cell wall; er: endoplasmatic reticulum; c: cytoplasm; v: vacuole. Scale bars: 1 µm. Arrows indicate PbDrk1-specific gold particles.

Although the lack of consensus in predicting subcellular localization in the in-silico analysis and confocal images does not demonstrate the cell wall localization of the protein, immunogold electron microscopy (EM) showed PbDrk1 is located in *P. brasiliensis* cell wall (Figure 4). Besides this, EM revealed protein localization in cytoplasm, vacuole, and endoplasmic reticulum (Figure 4). Together, our results showed a random distribution of Drk1 in *P. brasiliensis.*

### 3.4. Inhibition of Drk1 with Anti-PbDrk1 Antibody Alters the Interaction of P. brasiliensis with Pneumocytes and the Fungal Virulence in G. mellonella

**Figure 5:**
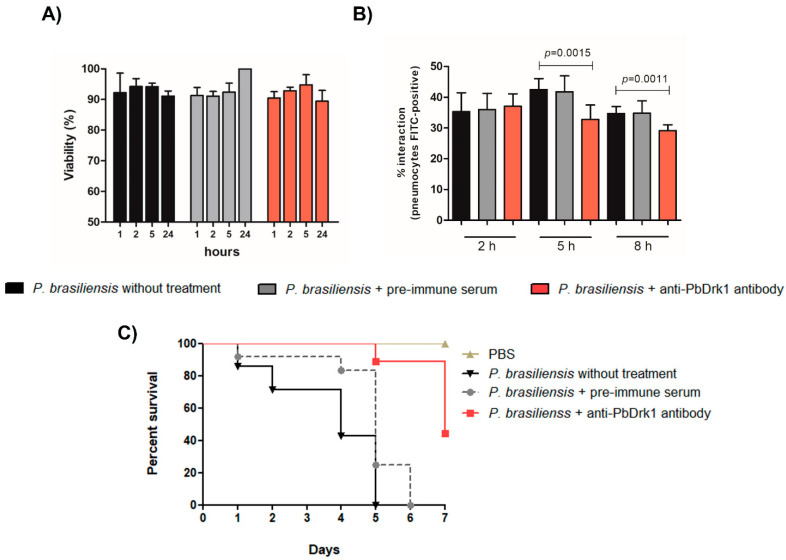
PbDrk1 inhibition and effects on *P. brasiliensis*-pneumocytes interaction and virulence. *P. brasiliensis* strain was treated with 20 µg mL^−1^ of anti-PbDrk1 antibody and as control groups we used *P. brasiliensis* treated with pre-immune serum (20 µg mL^−1^) or untreated. (**A**) Viability was evaluated for different times (1, 2, 5, and 24 h) by XTT assay. Results are presented as percentage of viability in comparison with the control (*P. brasiliensis* untreated). (**B**) *P. brasiliensis-*interaction with pneumocytes was analyzed 2, 5, and 8 h after the antibody′s treatment by flow cytometry. A total of 10,000 events were counted, and the percentage of pneumocytes-FITC positive (containing yeast cells) was determined. (**C**) Survival rate of infected *G. mellonella* (*n* = 10 larvae) infected with *P. brasiliensis* treated with the anti-PbDrk1 antibody, pre-immune serum control and untreated. Survival was evaluated for seven days. *P. brasiliensis* treated with anti-PbDrk1 antibody reduces fungus virulence, with a statistically significant difference compared to *P. brasiliensis* untreated (*p* < 0.0001).

The block of PbDrk1 was performed treating *P. brasiliensis* with anti-PbDrk1 antibody to evaluate the Drk1 importance to the *P. brasiliensis-*pneumocytes interaction and its virulence in *G. mellonella* model. The *P. brasiliensis* treatment with anti-PbDrk1 antibody in the same concentration used in the subsequent assays do not alter the fungal viability (Figure 5A).

Fungal treatment with anti-PbDrk1 antibody inhibits the interaction (adhesion/invasion) of *P. brasiliensis* with pneumocytes, particularly at 5 and 8 h of interaction, with a decrease of 1.3-fold and 1.1-fold, respectively (Figure 5B). Regarding the survival rate of *G. mellonella* (Figure 5C), the block of PbDrk1 led to a significant reduction in virulence, as seen in *P. brasiliensis* treated with the anti-PbDrk1 antibody that showed a survival rate of 44.4% at the end of experiment (7 days). Additionally, at 5 and 6 days of infection the anti-PbDrk1 treatment resulted in a survival rate of 88.8% compared to 0% of the untreated *P. brasiliensis* and treated with pre-immune serum control. Control group of larvae that received PBS showed 100% of survival at the end of the experiment (Figure 5C). The *P. brasiliensis* treatment with pre-immune serum showed no difference compared to *P. brasiliensis* without antibody treatment to viability, interaction ability, or virulence in *G. mellonella* (Figure 5).

### 3.5. PbDRK1 Knockdown Does Not Change the Growth Rate and Viability of P. brasiliensis

**Figure 6:**
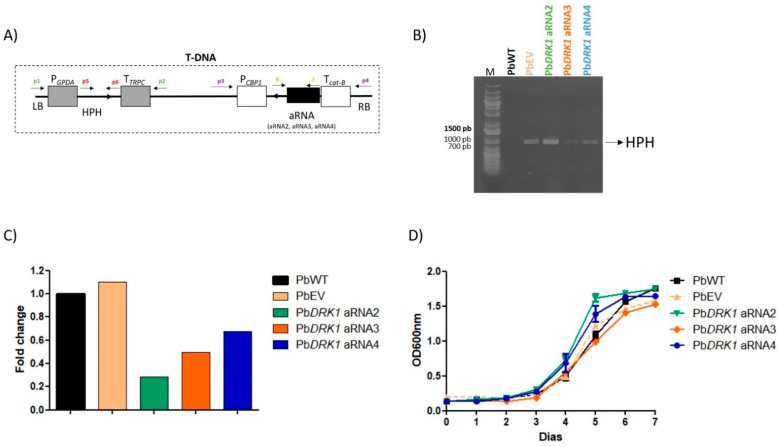
Knockdown of DRK1 of *P. brasiliensis.* (**A**) Transfer DNA (T-DNA) construct (carried out in the pUR5750 vector) for aRNA silencing of DRK1 via ATMT, containing the HPH (hygromycin B phosphotransferase) gene driven by the *Aspergillus nidulans* GPDA (glyceraldehyde 3-phosphate) promoter and TRPC (transcriptional terminator) with aRNA oligonucleotides under the control of CBP1 (calcium-binding protein) promoter from *H. capsulatum* and *cat*-B gene from *Aspergillus fumigatus.* (**B**) After selecting stable mitotic clones, hygromycin-resistant, three isolates (Pb*DRK1* aRNA2, Pb*DRK1* aRNA3, and Pb*DRK1* aRNA4) were selected, and the HPH gene amplification by PCR was performed to confirm the presence of T-DNA. (**C**) The relative DRK1 expression levels in the Pb*DRK1* aRNAs were determined by qRT-PCR compared to PbWT (that was set at 100%). The L34 reference gene was used as an endogenous control to normalize the relative DRK1 expression levels. (**D**) Growth of PbWT, PbEV, and Pb*DRK1* aRNA strains. Yeasts from all strains were cultivated at 37 °C for seven days, and the growth curve profiles were determined by reading the optical density at 600 nm every 24 h.

To perform a more in-depth evaluation of the relevance of Drk1, we verify whether its deficiency could influence some aspects of *P. brasiliensis* biology and virulence. To this proposal, antisense RNA (aRNA) methodology with ATMT was applied (Figure 6A). *P. brasiliensis* transformation was confirmed through the observation of the presence of HPH gene in the hygromycin-resistant clones (Figure 6B), indicating the presence of the T-DNA cassette in the genomic DNA. Then, the Drk1 transcripts quantification was evaluated by qRT-PCR in the three isolates of hygromycin-resistant strains (Pb*DRK1* aRNAs: *PbDRK1* aRNA2, Pb*DRK1* aRNA3, and Pb*DRK1* aRNA4) that revealed a 71.3, 50.3, and 32.5% lower expression than PbWT, respectively. This result confirmed the efficiency of the methodology in generating *P. brasiliensis* clones to down express DRK1. As expected, the control PbEV does not show a difference in DRK1 expression compared to the PbWT strain (Figure 6C). Despite the efficiency of the gene silencing used, the Pb*DRK1* aRNAs strains showed a similar growth rate in the BHI medium (1% glucose) (Figure 6D). Besides this, all strains had comparable viability at the time 0 and 168 h, at the beginning and end of the growth curve.

### 3.6. DRK1 Deficiency Alters the Morphology of P. brasiliensis, Resulting in Elongated Yeast Cells

**Figure 7:**
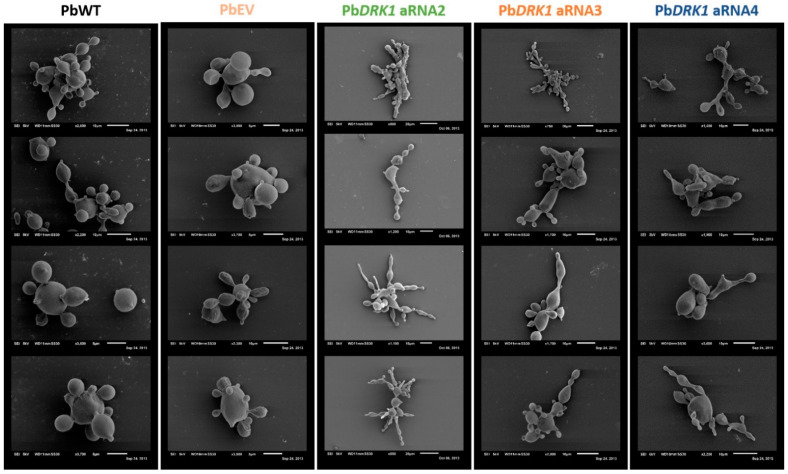
DRK1 is required for normal yeast morphology. The panel illustrates the morphological phenotype of PbWT, PbEV, Pb*DRK1* aRNA2, Pb*DRK1* aRNA3, and Pb*DRK1* aRNA4 yeast cells with scanning electron microscopy, all strains cultured for 72 h in liquid BHI medium (1% glucose) at 37 °C (150 rpm). Scale bars are indicated in each figure.

The strains were grown in BHI medium for 72 h, at 37 °C (150 rpm), to observe morphological alterations resulting from DRK1 silencing and then were examined by scanning electron microscopy. The Pb*DRK1* aRNA strains showed differences in the yeast morphology with pseudohyphal growth and mother cells more elongated. In addition, the Pb*DRK1* aRNA strain, which has lower Drk1 expression, better accentuated the described phenotype (Figure 7).

### 3.7. PbDRK1 Silence Affects the Virulence of P. brasiliensis in G. mellonella Model

**Figure 8:**
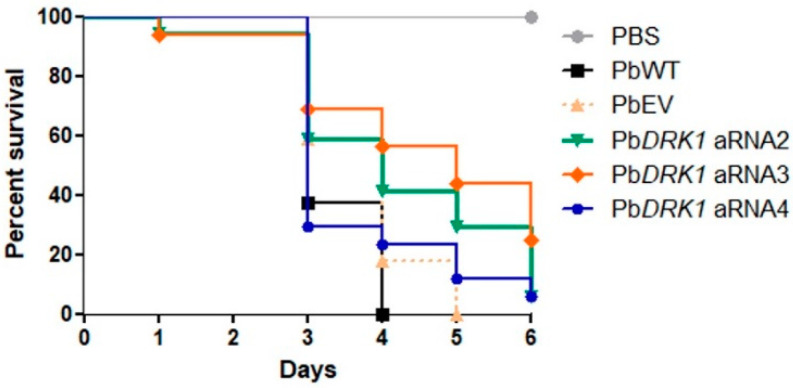
Knockdown of DRK1 expression confers a less virulent profile to *P. brasiliensis.* Survival curve of *G. mellonella* infected with 5 × 10^6^ cells of the strains, PbWT, PbEV, Pb*DRK1* aRNA2, Pb*DRK1* aRNA3, and Pb*DRK1* aRNA4, incubated at 37 °C, and the death monitored daily. PbWT vs. *PbDRK1* aRNA2, *p* = 0.0226; PbWT vs. Pb*DRK1* aRNA3, *p* = 0.0025; PbEV vs. *PbDRK1* aRNA3, *p* = 0.0185; at the end of experiment (*n* = 17 larvae).

The percentage of surviving larvae at day 4 after infection was 41.7, 56.2, and 23.52% to Pb*DRK1* aRNA2, Pb*DRK1* aRNA3, and Pb*DRK1* aRNA4, respectively, while infection with PbWT and PbEV resulted in 100 and 82.4% of death, respectively. Besides this, the virulence attenuation was more evident to Pb*DRK1* aRNA2 and Pb*DRK1* aRNA3, which shows the high degree of DRK1 silencing (Figure 8).

### 3.8. DRK1 Silencing Change the Chitin Level/Distribution in P. brasiliensis

**Figure 9:**
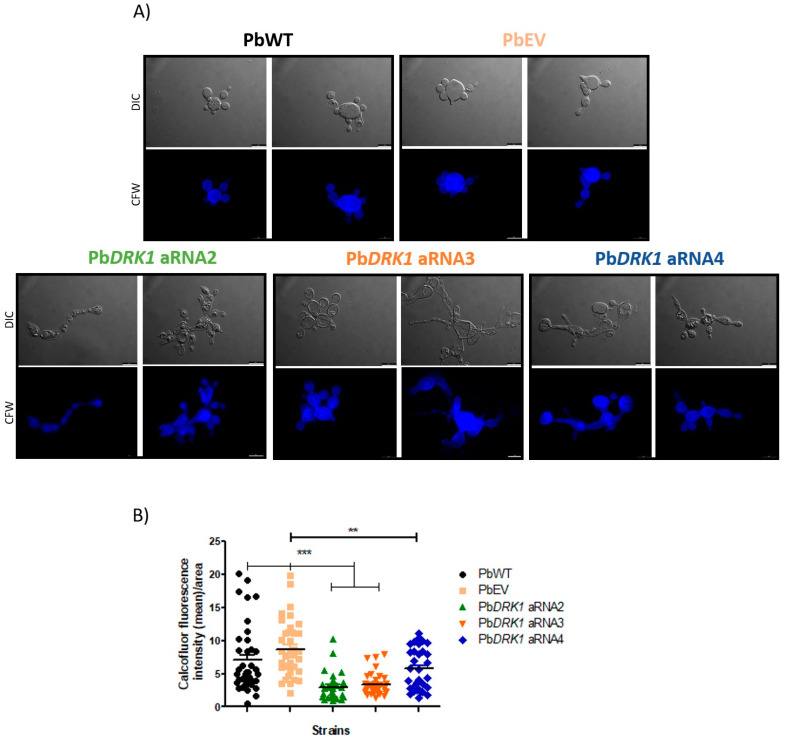
The level of DRK1 expression reduction correlates with a greater reduction of chitin in *P. brasiliensis*. Yeast cells of PbWT, PbEV, and PbDRK1 aRNA strains grown for 72 h at 37 °C in BHI medium (1% glucose) (150 rpm). Paraformaldehyde-fixed cells were stained with calcofluor white (50 µg mL^−1^) for 1 h at RT, and after several washes with PBS were (**A**) visualized under fluorescence microscopy, DMi8 (Leica) and images processed with LasAF software. Scale bars: 10 µm. (**B**) The chitin amount was estimated by measuring the calcofluor fluorescence intensity (mean)/area of each cell using ImageJ software. *** *p* < 0.0001 and ** *p* < 0.001.

Besides the morphological differences in Pb*DRK1* aRNAs yeast cells, comparing the chitin amount/distribution, it was possible to visualize an aberrant pattern of chitin distribution with some delocalization of the same along with the yeast cells (Figure 9A) with a reduction of chitin amount in *P. brasiliensis* cell wall, being this reduction 2.35-fold, 2.14-fold, and 1.26-fold to Pb*DRK1* aRNA2, Pb*DRK1* aRNA3, Pb*DRK1* aRNA4, respectively, compared to PbWT (Figure 9B). Furthermore, the greatest decrease in chitin correlated with the highest degree of DRK1 silencing.

### 3.9. Response to Osmotic Stress, Cell Wall Stressors and Antifungals Can Change due to Reduced Expression of Drk1 in P. brasiliensis

**Figure 10:**
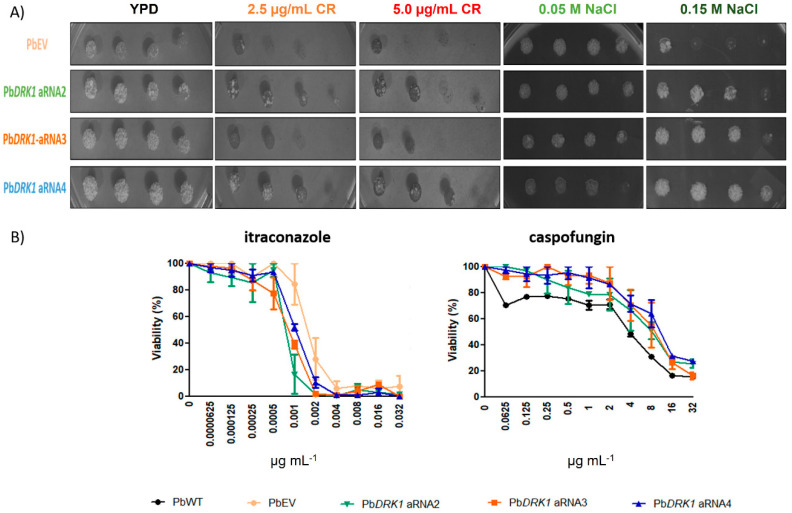
DRK1 down expression resulted in altered sensibility to osmotic, cell wall stressors, and antifungals. (**A**) Pb*DRK1* aRNA strains are more tolerant to the cell wall and osmotic stress. Ten-fold dilutions of cells were spotted onto YPD agar plates with the indicated concentrations of Congo red (CR) and NaCl. Plates were incubated at 37 °C for 7 days before being photographed. (**B**) DRK1 depletion leads to altered sensibility to itraconazole and caspofungin dose-response curves of antifungals against PbWT, PbEV, and Pb*DRK1* aRNA strains.

We observed that DRK1 silencing confers a phenotype more tolerant to Congo red (CR) and NaCl as shown in Figure 10A. The MIC was considered the lowest concentration to the antifungal susceptibility that resulted in a 50% reduction in fungal growth. Itraconazole PbEV showed a MIC of 0.002 µg mL^−1^ in contrast to 0.001 µg mL^−1^ for Pb*DRK1* aRNA strains; comparing the viability reduction to 0.0001 µg mL^−1^ of ITZ, it was possible to see a decrease of 15.6% to the PbEV, and 83.5%, 61.19%, and 48.2% to Pb*DRK1* aRNA2, aRNA3, and aRNA4 respectively, showing that the Drk1-silenced strains appear to be more sensitive to ITZ. In contrast, for caspofungin, PbWT showed a MIC of 4 µg mL^−1^ and the Drk1-silenced strains 16 µg mL^−1,^ demonstrating that the Drk1-silenced strains appear to be more tolerant to CSP (Figure 10B). Possibly these results suggest that cell wall composition alterations in the Pb*DRK1* aRNA strains may render it more resistant to damage by osmotic stress and cell wall disrupting agents.

## 4. Discussion

This report described the phenotypic effect of blockage and silencing of the *P. brasiliensis* Drk1 protein. The silencing of this gene in *B. dermatitidis, H. capsulatum, S. schenkii,* and *T. marneffei* suggested that Drk1 protein has a complex role in cell wall integrity, fungal morphogenesis, and virulence [1,20,22] and leads us to show the importance of this protein to *P. brasiliensis*. *P. brasiliensis* Drk1 shows high similarity, over 80%, among other fungi of Ascomycetes genus as *B. dermatitidis*, *H. capsulatum*, *S. schenckii*, and *Coccidioides immitis* [23]. In *P. brasiliensis*, Chaves et al. [23] demonstrated upregulation of this protein during the transition to yeast phase and osmotic stress, and more expressed in strains with a high virulent profile.

Our results showed *P. brasiliensis* Drk1 presence in several cell compartments as cytoplasm, vacuole, endoplasmic reticulum, and cell wall. Most hybrid histidine kinases (HHKs) are not predicted to be localized on the cell surface due to the lack of transmembrane domain [43,44]. However, Chaves et al. [23], through in silico analysis, indicated that PbDrk1 presents a signal peptide and possibly is an integral transmembrane protein that can explain its translocation to the cell surface in *P. brasiliensis*, as we observed in our microscopic analysis, where it can sense environment signals translating them into an intracellular input signals for others intracellular HHKs.

The fungal treatment with anti-Drk1 antibody reduced *P. brasiliensis* to interact with pneumocytes and reduced the virulence in *G. mellonella*, and we hypothesized that somehow Drk1 could act in the regulation of factors critical to the fungal-host interaction in *P. brasiliensis.* A less virulent profile was also observed in *G. mellonella* model to the Pb*DRK1* aRNA strains, confirming the importance of Drk1 to *P. brasiliensis* virulence. These findings are consistent with that observed for *B. dermatitidis, H. capsulatum,* and *S. schenckii*, in which Drk1-silencing markedly reduced pathogenicity [1,22]. An explanation can be that in *B. dermatitidis* and *H. capsulatum,* the DRK1-mutant have a reduction in the expression of essential adhesins, BAD-1 that is important adhesin to the interaction of *B. dermatitidis* with macrophages [1,45], and CBP-1 important to *H. capsulatum* intracellular survival and replication [1,46,47]. Besides this, for both species, a reduction in the expression of *AGS1* [1], a protein involved in the α-1,3 glucan synthesis and also fungal virulence, participates in the pathogenesis since it is able to hide the β-glucans avoiding the host cell detection [48]. Drk1 may sense host factors, triggering signaling pathway activation that results in gene expression modulation, mainly genes important to the host–fungal interaction.

*DRK1* downregulation did not alter the viability or growth rate of *P. brasiliensis;* however, it affects the yeast cell morphology. Morphological analysis revealed that *P. brasiliensis* DRK1 is essential to maintaining regular yeast shapes of cells; DRK1-silencing cells display a more elongated shape showing a morphology that resembles pseudohyphae. The same phenotype was also observed in *B. dermatitidis* and *H. capsulatum* [1]. The maintenance of yeast morphology is of great importance; the morphological change from mycelium to yeast and its maintenance is directly linked to virulence [1].

Our results showed that *P. brasiliensis* exposition to NaCl resulted in an altered pattern of Drk1 expression in the cells, with its homogeneous distribution tending in average conditions to an aggregated punctuation disposition in the cytoplasm. This phenomenon was also observed in *Candida guilliermondi* to Nik1 (homologous to Drk1) and can indicate that the protein could be associated with osmotic stress signaling [49].

*DRK1*-silenced strains are more tolerant to cell-wall perturbing agent Congo red and NaCl. This result is in contrast to that found for *B. dermatitis* in which DRK1-knockdown strain displays increased sensitivity to Congo red [1]; but similar to that found to the homologous NikA in *Aspergillus fumigatus*, with NikA mutation conferring resistance to the same cell wall stressor [50]. For *B. dermatitidis, DRK1*-silenced strain can respond to NaCl by Hog1 phosphorylation, indicating that Drk1 is not required to respond to salt stress [51], unlike to *P. marneffei* in which the DrkA deletion (homologous to Drk1) confers increased sensibility to NaCl [20]; indicating that Drk1 or its homologous plays roles that are not so conserved and therefore vary according to the species.

Chaves et al. [23] showed an increased sensibility to NaCl after *P. brasiliensis* treatment with iprodione, a fungicide known to inhibit class III of HHKs and therefore Drk1, activating constitutively the HOG pathway (but with the mode of action poorly understood); these data possibly are not comparable with those found in our study, in which Pb*DRK1* strains are more tolerant to osmotic stress, because recently it was demonstrated that fludioxonil, another fungicide, that also inhibits Drk1, shows its effects in the fungi changing the Drk1 behavior from kinase to phosphatase [51].

The increased resistance to Congo red in *P. brasiliensis DRK1*-silenced strains could be a consequence of considerable differences in polymer organization and cell wall content. The silencing of DRK1 in *P. brasiliensis*, rendered to the yeast cells a loss amount and different distribution of chitin, being this reduction correlated with the degree of silencing among the Pb*DRK1* aRNA strains, indicating that the Drk1 protein can participate in maintaining the cell wall integrity. An aberrant striated pattern of chitin in *P. marneffei* and *B. dermatitidis* DRK1-silenced strain was also observed [1,20]. In *S. cerevisiae* it has already been shown that mutant cells tolerant to Congo red exhibit a lower amount of chitin on the cell surface [52,53].

There is evidences reporting the caspofungin tolerance in laboratory strains and this has been associated as a cell wall salvage mechanism; with the fungi increasing the chitin production to compensate the decreased amount of β-1,3 glucan when glucan synthase is inhibited by this antifungal [54]. The mice infection with *C. albicans* in which the chitin synthesis was induced by calcium and calcofluor demonstrated that these cells are less susceptible to echinocandin compared to those with normal chitin amount [55]. In *C. albicans* the caspofungin treatment leads to the activation of Hog1p to control the oxidative and osmotic stresses that occur due to the exposure to this drug [56], and itraconazole also induced oxidative stress [57,58]. Therefore, we evaluated the susceptibility of *P. brasiliensis DRK1*-silenced strains to caspofungin and itraconazole, making it possible to assess whether changes in the silenced strains leads to a cell wall compensatory mechanism to balance its structure [54] or even to analyze the *DRK1*-silenced strain adaptation during the stress condition triggered by antifungals. It was possible to verify that *P. brasiliensis DRK1*-silenced strains seem to be more sensitive to itraconazole, indicating that possibly the oxidative stress triggered by this drug can be sensed by Drk1, and that the tolerance to caspofungin can occur in a way unrelated to the reduced amount of chitin. This question needs to be further evaluated.

## 5. Conclusions

In summary, our findings suggest the involvement of Drk1 in several aspects of *P. brasiliensis* such as maintenance of yeast cell morphology, cell wall composition, response to environmental stresses, and as a cell wall destabilizing drug, host–cell interaction, and virulence. However, several questions are worthy of further investigation such as what signals from the environment are sensed. Evaluation of potential receptors and downstream components that can interact with Drk1, to help in the elucidation of the mechanisms responsible for these changes that can shed light on the pathogenesis of *P. brasiliensis* at a molecular level, as well as the development of new therapies, once humans lack homologs to Drk1 needs to be done. This protein represents a potential target to treat PCM and other dimorphic fungal diseases.

## Figures and Tables

**Table 1:** Oligonucleotides used in this study.

Primers	Sequences	References
DRK1-F	5′-CATATGACTCGTGGTGAT-3′	Current work
DRK1-R	5′-GCGGCCGCGCACAGAGACAG-3′	Current work
DRK1AS2-F	5′-CCGCTCGAGCGGCTACCAACGGCGATGTGAC-3′	Current work
DRK1AS2-R	5′-GGCGCGCCCACAAATGATTGCATGGTC-3′	Current work
DRK1AS3-F	5′-CCGCTCGAGCGGCCTCCATCTCGACGATCT -3′	Current work
DRK1AS3-R	5′-GGCGCGCCTGTAGTTTGTGTCAGACT-3′	Current work
DRK1-AS4-F	5′-CCGCTCGAGCGGTGACACAAACTACAAAGA-3′	Current work
DRK1-AS4-R	5′-GGCGCGCCTCATCAATTTTCACCCTC--3′	Current work
Hph-F	5′-AACTCACCGCGACGTCTGTCGA-3′	[27]
Hph-R	5′-CTACACAGCCATCGGTCCAGA-3′	[27]
DRK1-qPCR-F	5′-CTTTTCCCTTTGGCGGCTACAA-3′	Current work
DRK1-qPCR-R	5′-ACTATCCTCCGGATTAGGTCTTCTAAC-3′	Current work
L34-F	5′-TCAATCTCTCCCGCGAATCC-3′	[28]
L34-R	5′-AGTTGGCGATTGTTGTGCGG-3′	[28]

## Data Availability

The raw data supporting the conclusions on this article will be made available by the authors, without undue reservations, but all relevant data are within the paper.

## Supplementary Materials

The following are available online at https://www.mdpi.com/article/10.3390/jof7100852/s1. Figure S1. PbDrk1 identity confirmation. Mascot results. Peptide summary report of identified protein. Matched peptides are highlighted in red, in which its sequence coverage reaches 59% of the Drk1 sequence of *P. brasiliensis.*
