# Supplementary material for: Drk1, a Dimorphism Histidine Kinase, Contributes to Morphology, Virulence, and Stress Adaptation in Paracoccidioides brasiliensis
Source: J Fungi (Basel). 2021 Oct 12;7(10):852. doi: 10.3390/jof7100852 (PMC8539220; doi:10.3390/jof7100852)
Supplement: Supplementary file 1 [file jof-07-00852-s001.zip › jof-1369358-supplementary.pdf]

{MATRIX SCIENCE} Mascot Search Results

Protein View

Match to: **EEH42759.2** Score: **3021**
hypothetical protein PADG_07579 [Paracoccidioides brasiliensis Pb18]
Found in search of 281474976710778.mgf

Nominal mass (M_r): **142545**; Calculated pI value: **5.34**
NCBI BLAST search of EEH42759.2 against nr
Unformatted sequence string for pasting into other applications

Fixed modifications: Carbamidomethyl (C)
Variable modifications: Oxidation (M)
Cleavage by Trypsin: cuts C-term side of KR unless next residue is P
Sequence Coverage: **59%**

Matched peptides shown in **Bold Red**

```
   1 MTRGDETLLA VAGILQGLAK DVPNSASFPF GGYKANNSTN GDVTKIKLPG
  51 EESDGKAVLE HELEDLIRRI VTMQSFVPPS RRSTRAPNFA SGQHSDLEGS
 101 FKSLPESDTN YKEDIQFLLN RVQLQAQEIQ LQKDVISKVR EELRNQEKRT
 151 EEALGRVKID DVSILERELR KHQQANEAFQ KALREIGGII TQVANGDLSM
 201 KVQIHPLEMD PEITTFKRTI NTMMDQLQVF GSEVSRVARE VGTEGILGGQ
 251 AQISGVHGIW KELTENVNIM AKNLTDQVRE IATVTTAVAH GDLSQKIESQ
 301 AKGEILELQQ TINTMVDQLR TFAIEVNRVA KDVGIEGVLG GQAQIDGVQG
 351 KMNELTVNVN AMAENLTTQV RDIAMVTTAV AKGDLTQKVQ ANCKGEILAL
 401 KTIINSMVDQ LKQFAQEVTK IAKEVGTDGV LGGQATVHDV EGTWKDLTEN
 451 VNGMAMNLTT QVREIADVTT AVAKGDLTKK VTADVKGEIL DLKNTINGMV
 501 DRLNTFAFEV SKVAREVGTD GTLGGQAKVD NVEGKWKDLT DNVNTMAQNL
 551 TSQVRGISDV TQAIAKGELS KKIEVHAQGE ILTLKVTINN MVDRLANFAH
 601 ELKRVARDVG VDGKMGGQAN VEGISGRWKE ITEDVNTMAE NLTSQVRAFG
 651 EITDAATDGD FTKLITVNAS GEMDELKRKI NKMVSNLRDS IQRNTAAREA
 701 AELANRTKSE FLANMSHEIR TPMNGIIGMT QLTLDTDDLK PYPREMLNVV
 751 HSLANSLLTI IDDILDISKI EANRMVIERI PFSMRGAVFN ALKTLAVKAN
 801 EKILNLIYQV DSSIPDFVTG DPFRLRQIIL NLVGNAIKFT ERGEVRVTIL
 851 KSDREECRPN EYSFEFIVSD TGIGIEEDKL DLIFDTFQQA DGSTTRKFGG
 901 TGLGLSISKR LVNLMGGDVW VTSEFGHGSK FHFTCVVEMA DQDISNIAAS
 951 LLPYKNHRVL FIDRGETGGY AKEITNMLKQ LDLDPIVVTN ESQIPPPEIQ
1001 DSSGKDSGHA YDVIIVDSVN TAKSLRTYDE FKYIPIVLLC PVVSVNLKSA
1051 LDLGITSYMT TPCLPVDLGN GMIPALEGRS TPITTDHTRS FDILLAEDND
1101 VNQRVAVKIL EKCNHDVTVV SNGLQALEAI KQRRFDVILM DVQMPVMGGF
1151 EATGKIREYE REHGLPRTPI IALTAHAMLG DREKCIQAQM DEYLAKPLKH
1201 NQMVQMILKC VTLGDSLLEK SNEPSALGGG DDTHHLTRHT LVQSSEARSQ
1251 RLLMDSRALA AFGSSGANHG LNRKPDMVST VRFPCDQLLS LC
```

Figure S1. PbDrk1 identity confirmation. Mascot results. Peptide summary report of identified protein. Matched peptides are highlighted in red, in which its sequence coverage reaches 59% of the Drk1 sequence of *P. brasiliensis*.
